# Thrombotic Storm: A Challenging Case of Catastrophic Antiphospholipid Syndrome

**DOI:** 10.7759/cureus.100139

**Published:** 2025-12-26

**Authors:** Ana Filipa Silva, Mari Mesquita, Joana Gomes, Daniela Barbosa, Lindora Pires

**Affiliations:** 1 Internal Medicine, Unidade Local de Saúde Tâmega e Sousa, Penafiel, PRT

**Keywords:** abdominal pain, acute diarrhea, autoimmune disease, caps, hypercoagulability, multiorgan ischemia, sjögren’s syndrome

## Abstract

We present the clinical case of a 68-year-old woman with a history of cerebrovascular disease, primary Sjögren’s syndrome, and suspected antiphospholipid syndrome, admitted for acute diarrhea and fever. She showed good clinical and laboratory improvement under ciprofloxacin until the fifth day of hospitalization, when she developed abdominal pain with guarding, fever, and vomiting. Laboratory tests revealed elevated inflammatory markers and rhabdomyolysis. An abdominopelvic CT scan showed multiorgan ischemia, raising the suspicion of catastrophic antiphospholipid syndrome (CAPS).

## Introduction

Catastrophic antiphospholipid syndrome (CAPS) is a rare and life-threatening manifestation of antiphospholipid syndrome (APS), characterized by severe thrombotic complications, usually involving the microvasculature as well as large vessels, affecting multiple organs simultaneously or within a short period of time. Risk factors for catastrophic APS include infection, surgery, active malignancy, estrogen therapy, pregnancy/puerperium, and systemic lupus erythematosus (SLE).

The organs most frequently affected in catastrophic antiphospholipid syndrome (CAPS) include the kidneys (74%), brain (56%), lungs (55%), heart (53%), and skin (45%). Other commonly involved sites are peripheral vessels (37%), liver (34%), and the gastrointestinal tract (12%). This multisystem involvement reflects the widespread microvascular thrombosis characteristic of the syndrome.

CAPS occurs in approximately 1% of patients diagnosed with APS [1] and is associated with a high mortality rate. A prior diagnosis of APS is present in nearly 50% of CAPS cases. CAPS shows a female predominance, with roughly 70% of cases occurring in women. In patients without systemic lupus erythematosus, the sex distribution tends to be more equal. The condition has been reported across a wide age spectrum, from pediatric to geriatric populations. CAPS carries a high fatality rate if left untreated. Even with aggressive intervention, mortality can exceed 30%. Early recognition and prompt initiation of appropriate therapies are critical to improving survival. The leading causes of death include cerebrovascular events (such as stroke, intracerebral hemorrhage, and encephalopathy), cardiac complications, and infections, together accounting for approximately two-thirds of all related deaths. The presence of SLE [2,3] was associated with an increased risk of mortality.

By definition, CAPS is characterized by the simultaneous involvement of multiple organ systems, driven by a widespread thrombotic process affecting the microvasculature and, in some cases, both microvascular and macrovascular territories.

The pathophysiological mechanism underlying the development of a “thrombotic storm” as opposed to isolated thrombosis in a single vascular territory remains poorly understood. It is currently unknown whether patients with CAPS exhibit antiphospholipid antibodies (aPL) with distinct antigen specificity, avidity, titers, or other immunological properties compared to those observed in classic APS. The majority of patients diagnosed with CAPS demonstrate triple aPL positivity, typically with high-titer immunoglobulin G (IgG) anticardiolipin and anti-β2 glycoprotein I (anti-β2GPI) antibodies. Antiphospholipid antibodies (aPL) induce activation of vascular endothelial and immune cells [4], resulting in the upregulation of surface adhesion molecules and the release of proinflammatory cytokines, procoagulant factors, and extracellular vesicles, along with other cell-derived microparticles. Several preclinical studies have implicated complement activation as a contributing factor in the thrombotic manifestations of both APS and CAPS. Upon activation, the complement cascade generates C5a (a potent anaphylatoxin) and the membrane attack complex (MAC, C5b-9) [5], which directly injures cell membranes. The lytic activity of C5b-9 induces hemolysis and the release of free heme, a highly prothrombotic molecule. In addition, complement-mediated endothelial injury exposes subendothelial collagen and tissue factor, thereby promoting platelet activation and initiation of the coagulation cascade. Complement-mediated neutrophil activation can also result in the extrusion of neutrophil extracellular traps (NETs) [6,7], further amplifying thrombosis.

## Case presentation

We report the case of a 68-year-old woman with a medical history of arterial hypertension, class I obesity, primary Sjögren’s syndrome with extraglandular manifestations, depressive disorder, and cerebrovascular disease.

The patient had been admitted two months prior to the Internal Medicine Department due to an ischemic stroke in the left thalamocapsular region. Contrast-enhanced brain MRI revealed two acute ischemic lesions in the left thalamocapsular area, as well as multiple chronic ischemic foci in the periventricular and subcortical regions of both cerebral hemispheres. The etiological investigation of the ischemic event raised suspicion for antiphospholipid antibody syndrome (APS). The reported antiphospholipid antibody profile in this patient showed a strong lupus anticoagulant, elevated anti-β2 glycoprotein I (β2GPI) IgG, and normal anticardiolipin levels. Although most CAPS patients are described as having “triple positivity,” it is well recognized that CAPS can also occur in non-triple-positive APS phenotypes. Therefore, the patient represents a biologically typical but non-triple-positive CAPS presentation. Emerging evidence suggests that antibodies outside the classical criteria, such as anti-prothrombin/phosphatidylserine (anti-PT/PS) IgM, may contribute to thrombosis risk and could serve as additional pathogenic markers in APS. Several recent studies have highlighted the potential role of these non-criteria antibodies in precipitating thrombotic storms, supporting the concept that CAPS can develop even in patients who do not meet the conventional “triple-positive” serologic profile. The patient was referred to the Rheumatology Department with a recommendation to repeat the APS serologic panel 12 weeks after the initial event (Table 1).

**Table 1:** Autoimmune panel

Parameter	Results	Reference value
Lupus anticoagulant	Positive	-
Complement C3	106 mg/dL	90-180 mg/dL
Complement C4	<5 mg/dL	10-40 mg/dL
Erythrocyte sedimentation rate	34 mm	0-20 mm
Anti-prothrombin/phosphatidylserine (anti-PT/PS) IgG	14.6 U/mL	<30 U/mL
Anti-prothrombin/phosphatidylserine (anti-PT/PS) IgM	90 U/mL	<30 U/mL
Anti-β2 glycoprotein I (anti-β2GPI) IgM	1.60 U/mL	<7 U/mL
Anti-β2 glycoprotein I (anti-β2GPI) IgG	16 U/mL	<7 U/mL
Anticardiolipin IgM	4.6 MPL-U/mL	<10 MPL-U/mL
Anticardiolipin IgG	5.3 GPL-U/mL	<10 GPL-U/mL
IgA	247 mg/dL	70-400 mg/dL
IgG	1132 mg/dL	700-1600mg/dL
IgM	181 mg/dL	40-230 mg/dL
IgG4	29.7 mg/dL	8-140 mg/dL
Antinuclear antibody test (ANA)	38	<0.7

Two weeks prior to the scheduled consultation, the patient presented to the Emergency Department with a three-day history of watery diarrhea (seven episodes per day), without blood or mucus. The condition was associated with fever (maximum axillary temperature: 38.6 ºC) that had begun one day earlier. She denied abdominal pain, nausea, or vomiting. No other complaints were reported. There were no sick contacts and no history of consumption of unpasteurized foods, raw or undercooked meat or fish. She also denied intake of vitamin or mineral supplements such as magnesium, recent antibiotic use, laxative use, or recent travel.

On physical examination in the Emergency Department, the patient was awake but slow to respond and opened her eyes only to verbal stimuli. Her speech was unintelligible, and she had evident xerosis. She was able to follow simple commands. Muscle strength was globally reduced, consistent with a prostrated state, with decreased strength in the right upper limb (Grade 4+), in keeping with a prior ischemic stroke. No obvious sensory deficits were noted. The patient appeared pale and dehydrated. Cardiac auscultation revealed S1 and S2 to be rhythmic without audible murmurs. Pulmonary auscultation showed symmetrical vesicular breath sounds without adventitious sounds. The abdomen was soft and depressible, without tenderness or guarding, and there were no signs of peritoneal irritation or palpable organomegaly. She was febrile (temperature 38.9 ºC), with a blood pressure of 108/62 mmHg, heart rate of 115 bpm, and peripheral oxygen saturation of 99% on room air.

The patient was subsequently admitted to the Internal Medicine Department with a presumptive diagnosis of infectious colitis, and antibiotic therapy with ciprofloxacin was initiated. She showed favorable clinical evolution up to the fifth day of hospitalization with sustained apyrexia, a downward trend in inflammatory markers, and complete resolution of diarrheal symptoms. No infectious agent had been isolated up to that point.

On the fifth day of hospitalization, she developed new-onset diffuse abdominal pain accompanied by four episodes of watery vomiting and a recurrence of fever. At that time, the patient reported pain intensity of 8/10. The abdomen was distended, with no visible skin lesions. No visible masses or signs of collateral circulation were noted. Respiratory abdominal motion was normal. Bowel sounds were present with normal pitch and frequency, and no vascular bruits were heard. Hepatic span measured 7 cm. Diffuse tympanism was present, with no shifting dullness or fluid wave. The abdomen was tender on both superficial and deep palpation, with associated guarding. No signs of peritoneal irritation were observed. Arterial blood gas analysis reveals metabolic acidosis with concomitant hyperlactacidemia. The analytical results from that time are described in Table 2.

**Table 2:** Analytical panel during exacerbation

Parameter	Results	Reference value
Hemoglobin	11.7 g/dL	12-15 g/dL
Leukocytes	22900	4500-11000
Platelets	109000	150000-400000
Urea	108 mg/dL	10-50 mg/dL
Creatinine	2.14 mg/dL	0.66-1.09 mg/dL
Sodium	128 mmol/L	135-145 mmol/L
AST (Aspartate Aminotransferase)	154 UI/L	10-31 UI/L
ALT (Alanine Aminotransferase)	110 UI/L	10-31 UI/L
LDH (Lactate Dehydrogenase)	642 UI/L	135-225 UI/L
CPK (Creatine Phosphokinase)	1107 UI/L	10-149 UI/L
C-reactive protein (CRP)	257.1 mg/L	<5 mg/L

In the context of acute abdomen, recurrent fever and vomiting in a patient hospitalized for acute diarrhea and undergoing antibiotic therapy with ciprofloxacin, the following differential diagnoses were considered: intra-abdominal abscess, toxic megacolon, pancreatitis, gastroenteritis caused by a pathogen unresponsive to antibiotic therapy, gastrointestinal perforation, intestinal ischemia, and bowel obstruction.

**Figure 1:**
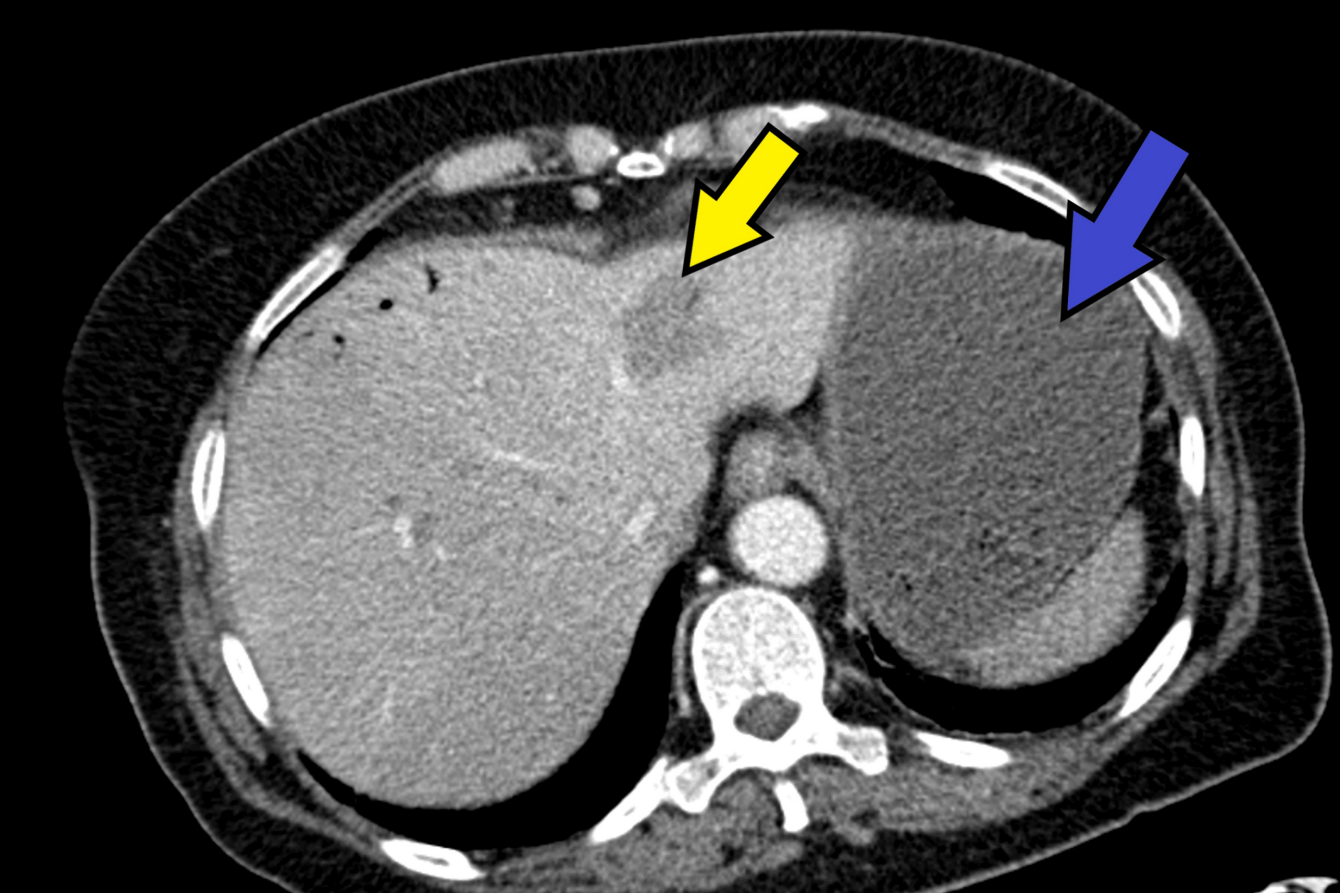
Contrast-enhanced abdominopelvic CT scan Blue arrow: Splenic infarcts; Yellow arrow: Presence of portal venous gas involving segments IV and VIII of the liver

**Figure 2:**
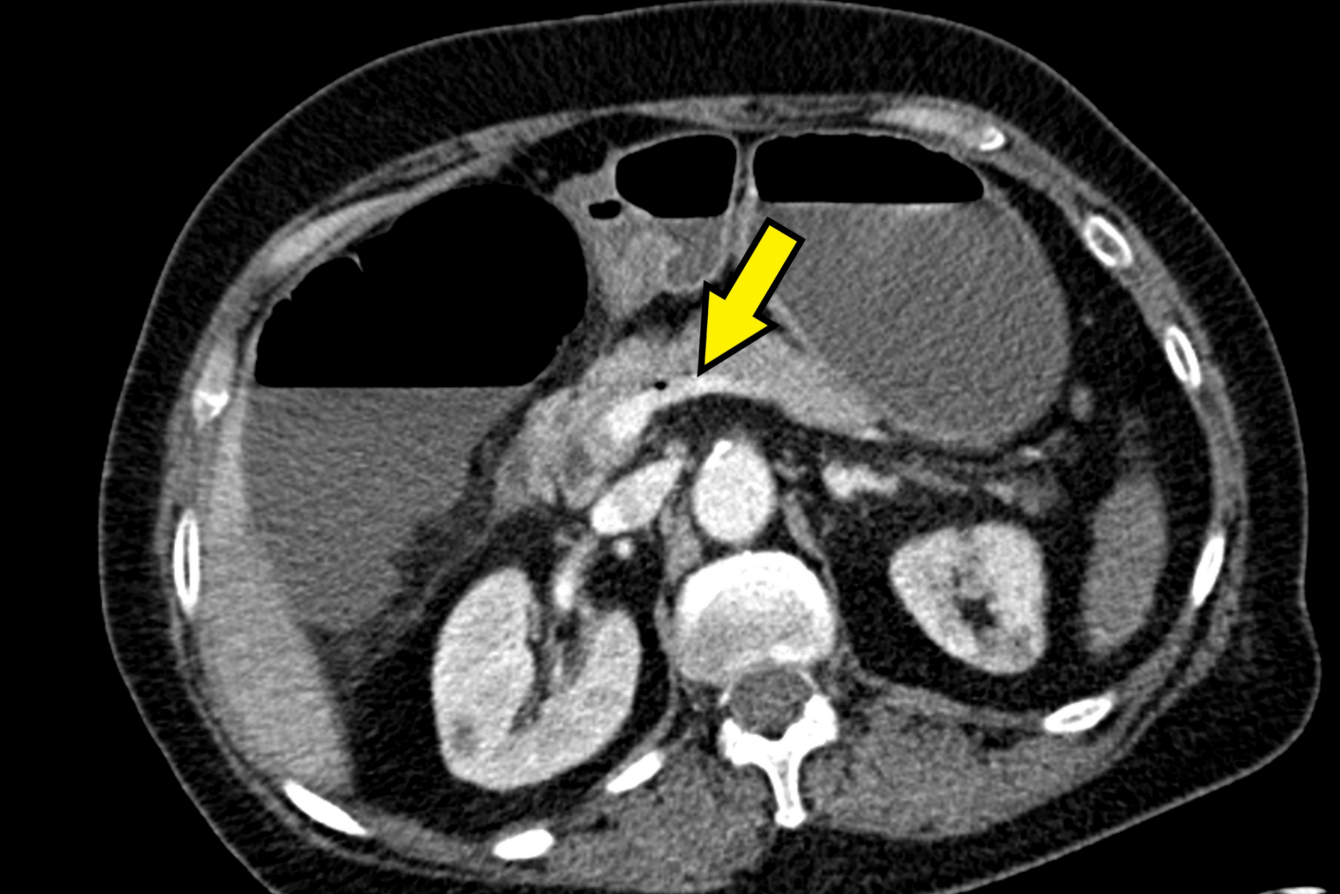
Contrast-enhanced abdominopelvic CT scan Yellow arrow: Signs of mesenteric ischemia with intestinal pneumatosis

**Figure 3:**
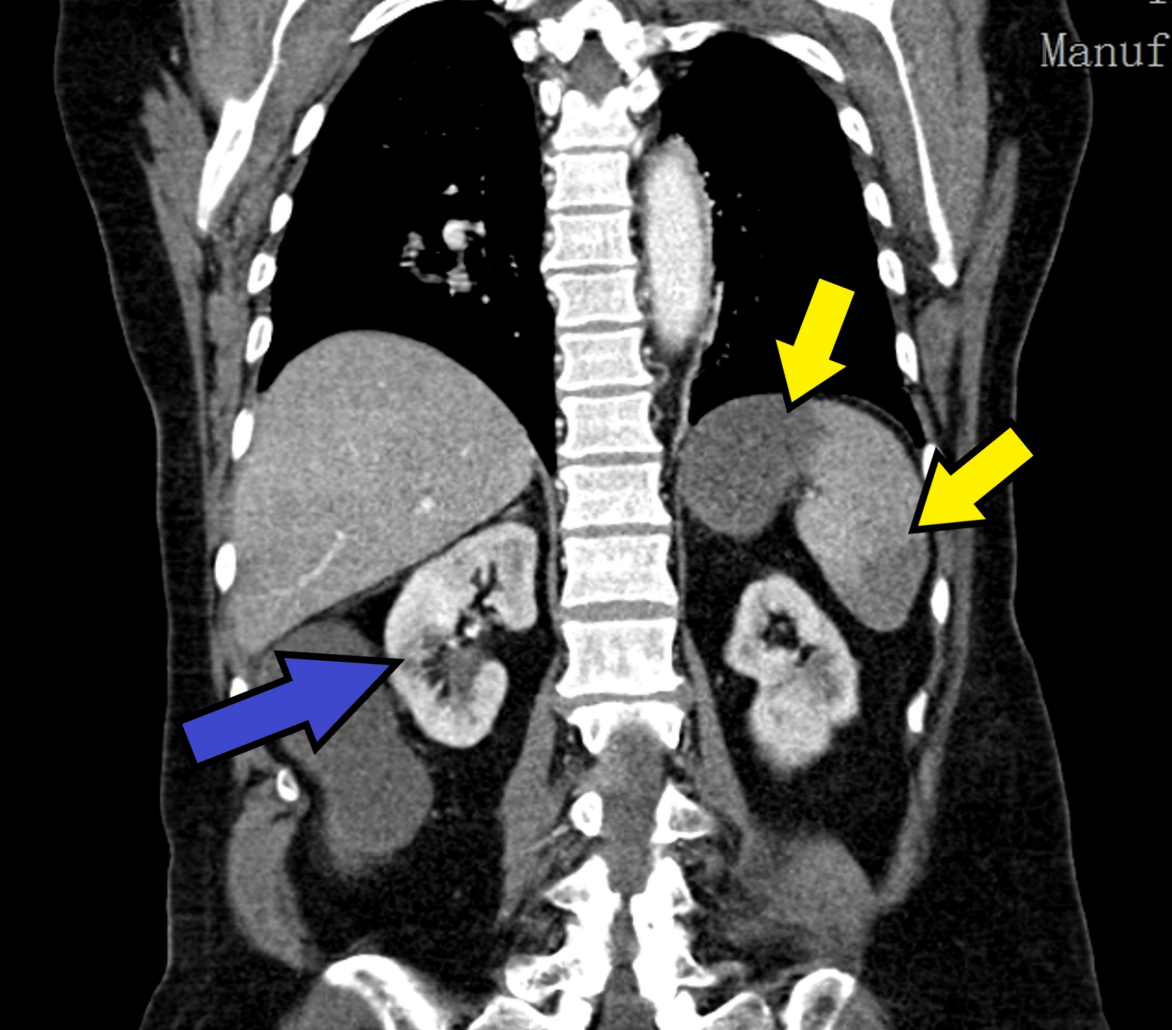
Contrast-enhanced abdominopelvic CT scan Yellow arrow: Splenic infarcts; Blue arrow: Renal infarcts

Abdominopelvic CT angiography demonstrated findings suggestive of mesenteric ischemia involving the cecum, descending colon, and several ileal loops in the pelvis, with evidence of intestinal pneumatosis. Multiple splenic infarcts were identified, affecting approximately 50% of the splenic parenchyma, along with infarcts in both kidneys and in the left and right lobes of the liver. Additionally, hepatic portal venous gas was observed, involving segments IV and VIII of the liver, with small air bubbles present within the portal vein. Figures 1-3 illustrate the changes described in CT.

The hypothesis of probable CAPS was thus considered, given the positivity for lupus anticoagulant and ischemia involving four organs (colon, kidney, liver, and spleen) with simultaneous manifestation.

Urgent evaluation by General Surgery was requested. The patient underwent right hemicolectomy and segmental jejunal enterectomy and was admitted to the Intensive Care Unit (ICU). She underwent right hemicolectomy and segmental jejunal enterectomy and was admitted to the ICU, dying that night. 

Subsequent histological examination of the right hemicolectomy specimen confirmed ischemic necrosis involving the colonic segment, terminal ileum, and ileocecal appendix, predominantly affecting the mucosa and submucosa. This was accompanied by a primarily transmural inflammatory infiltrate. The surgical resection margins also showed evidence of ischemic injury.

## Discussion

CAPS is a rare and life-threatening manifestation of APS, characterized by severe thrombotic complications, usually affecting the microvasculature as well as large vessels, involving multiple organs simultaneously or within a short period of time. Risk factors for catastrophic APS include infection, surgery, active malignancy, estrogen therapy, pregnancy/postpartum, and systemic lupus erythematosus SLE.

In this patient’s case, the ischemic stroke two months earlier was likely the first manifestation of antiphospholipid syndrome (APS). Between the initial hospitalization [7] and while awaiting the Rheumatology consultation, the patient did not initiate anticoagulant therapy.

The patient had a high-risk APS profile, including a prior ischemic stroke, strong lupus anticoagulant, and positive anti-β2 glycoprotein I antibodies. Despite this, anticoagulation was not initiated while awaiting rheumatology follow-up. This represents a critical gap in secondary thromboprophylaxis that likely contributed to the subsequent thrombotic complications. Current European Alliance of Associations for Rheumatology (EULAR) recommendations [8] advise long-term anticoagulation after a first thrombotic event in patients with antiphospholipid syndrome, particularly in those with a high-risk antiphospholipid antibody profile, to prevent recurrent thrombosis. Highlighting this missed opportunity underscores the importance of timely prophylactic intervention and strengthens the educational value of the case, providing a clear systems-based lesson for clinicians managing APS patients.

During the current hospitalization for presumed infectious colitis, the patient initially exhibited a favorable clinical course under ciprofloxacin therapy. However, clinical deterioration occurred on the fifth day of admission. In the absence of definitive microbiological confirmation, the infection should be considered a suspected trigger of CAPS. Notably, systemic inflammation itself, even without a proven infectious etiology, may act as a precipitating factor for CAPS. Inflammatory states can promote endothelial activation, complement cascade amplification, and NET formation, all of which play a central role in the thromboinflammatory pathogenesis of CAPS.

CAPS is a rare and potentially life-threatening manifestation of antiphospholipid syndrome, characterized by disseminated small-vessel thrombosis with rapid multiorgan involvement. The classical classification criteria proposed by Asherson et al. [9], widely used in clinical practice and in the CAPS Registry [1], include: (1) involvement of three or more organs, systems, or tissues; (2) development of clinical manifestations within one week; (3) histopathological confirmation of small-vessel thrombosis; and (4) presence of antiphospholipid antibodies. Fulfillment of all these criteria defines definite CAPS, whereas the absence of one criterion allows classification as probable CAPS. It is important to note that these criteria were primarily developed for research classification rather than rigid diagnostic purposes. In clinical practice, CAPS diagnosis is often made based on a highly suggestive clinical picture, even in the absence of histopathological confirmation or persistent antiphospholipid antibody positivity, limitations that are common in acute, rapidly progressive disease with high early mortality.

Similarly, the 2023 American College of Rheumatology (ACR)/EULAR classification criteria for antiphospholipid syndrome were designed to standardize patient selection for research studies and require greater stringency, including persistent seropositivity confirmed on two occasions at least 12 weeks apart [10]. However, strict application of these criteria may be impractical in the context of CAPS, where the fulminant clinical course frequently precludes repeat antibody testing. It should also be noted that many published CAPS cases, including those in the CAPS Registry, rely on single-time-point antiphospholipid antibody positivity when the clinical context is strongly suggestive [1]. Therefore, although the present case does not fully meet the 2023 ACR/EULAR classification criteria due to the absence of persistent serology, the clinical presentation is highly indicative of CAPS, supporting its classification as probable CAPS in a real-world clinical setting [10].

This patient meets all the required clinical criteria for catastrophic antiphospholipid syndrome, except for the laboratory confirmation of antiphospholipid antibodies at least six weeks apart, which could not be obtained due to the patient’s early death. Therefore, in the absence of this fourth criterion, the case is classified as probable CAPS (Table 3) [10].

**Table 3:** Classical (Asherson) versus 2023 ACR/EULAR CAPS criteria; definite and probable categories with correspondence to the current case CAPS: catastrophic antiphospholipid syndrome; aPL: antiphospholipid antibodies; ACR/EULAR: American College of Rheumatology/European Alliance of Associations for Rheumatology Asherson et al. [9]; ACR/EULAR [10]

Feature	Classical CAPS criteria (Asherson)	ACR/EULAR 2023 criteria	Present case
Purpose	Classification/clinical guidance	Research classification	—
Multiorgan involvement	≥3 organs	Scored clinical domains	✔
Rapid onset	<1 week	Integrated into clinical domains	✔
Histopathology	Required (definite)	Not mandatory	✔
Persistent aPL (≥12 weeks)	Ideal but not mandatory	Required	✘
Final classification	Definite/probable CAPS	May not classify as APS	Probable CAPS

Initial therapeutic management should be aggressive and based on three fundamental pillars: anticoagulation, glucocorticoids, and therapeutic plasma exchange (TPE) or intravenous immune globulin (IVIG). The choice of initial anticoagulant is intravenous unfractionated heparin. Regarding corticosteroid therapy, high-dose corticosteroids are recommended, with 1 g of methylprednisolone daily for at least a three-day period. In most patients with CAPS, TPE or IVIG is recommended (in addition to anticoagulation and high-dose glucocorticoids), although both therapies are generally not used simultaneously.

A 2018 report involving 471 patients observed that triple therapy, comprising anticoagulation, glucocorticoids, and either plasma exchange or intravenous immune globulin (IVIG), was associated with improved survival outcomes (triple therapy: 71% survival rate; single or dual therapy: 59% survival rate; no therapy: 25% survival rate) [11].

CAPS carries a high fatality rate if left untreated. Even with aggressive management [12,13], mortality can exceed 30%. Early recognition and prompt initiation of appropriate therapy are essential and may significantly improve patient outcomes.

The main causes of death in CAPS are cerebrovascular events (such as stroke, intracerebral hemorrhage, and encephalopathy), cardiac complications, and infections, which together accounted for approximately two-thirds of fatalities in one analysis [13]. In the same study, the presence of systemic lupus erythematosus (SLE) was associated with increased mortality.

Individuals who recover from an episode of CAPS remain at risk for disease recurrence. In an observational study of 58 patients who survived an initial CAPS episode and were followed for an average of 67 months, approximately 20% experienced recurrent APS-related thromboembolic events, although no cases of recurrent CAPS were reported [14]. Notably, 40% of these recurrent events occurred during the perioperative period. This high incidence of perioperative thromboembolic complications underscores the importance of minimizing time off anticoagulation in these patients when undergoing surgical procedures.

Although triple therapy (anticoagulation with heparin, corticosteroids, and either plasma exchange or IVIG represents the standard of care for CAPS, it was not initiated urgently in this patient. Several real-world barriers contributed to this delay. First, diagnostic uncertainty arose due to the non-triple-positive serology and the need to differentiate CAPS from other causes of acute multiorgan dysfunction. Second, the patient experienced rapid clinical deterioration, limiting the window for safe initiation of full therapy. Third, urgent surgical considerations related to ischemic complications necessitated prioritization of operative management over immediate immunomodulatory interventions. Recognizing these practical constraints illustrates the challenges of implementing guideline-based CAPS therapy in real-world settings and highlights the importance of systems-based learning for timely recognition and management.

## Conclusions

This case illustrates the fulminant course and diagnostic complexity of CAPS, a rare but potentially fatal condition. In our patient, the lack of early anticoagulation following an initial ischemic event and the presence of an infectious trigger may have contributed to the development of CAPS. Although the clinical, radiologic, and histopathologic findings supported a diagnosis of CAPS, the inability to complete serologic confirmation due to the patient’s rapid clinical deterioration limited the classification to probable CAPS.

The case underscores the critical importance of early recognition of APS, prompt initiation of appropriate prophylactic anticoagulation in high-risk patients, and a high index of suspicion for CAPS in the setting of acute multiorgan ischemia. Timely implementation of triple therapy (anticoagulation, high-dose corticosteroids, and plasma exchange or intravenous immunoglobulin) remains the cornerstone of management and has been associated with improved survival. Despite advances in treatment, mortality remains high, emphasizing the need for increased clinical awareness, especially in patients with incomplete but suggestive features of CAPS.
